# Insulin-Like Growth Factor-1 but Not Insulin Predicts Cognitive Decline in Huntington’s Disease

**DOI:** 10.1371/journal.pone.0162890

**Published:** 2016-09-14

**Authors:** Linda Salem, Nadine Saleh, Gaelle Désaméricq, Katia Youssov, Guillaume Dolbeau, Laurent Cleret, Marie-Laure Bourhis, Jean-Philippe Azulay, Pierre Krystkowiak, Christophe Verny, Françoise Morin, Stéphane Moutereau, The French Huntington Study Group, Anne-Catherine Bachoud-Lévi, Patrick Maison

**Affiliations:** 1 Université Paris Est, Faculté de médecine, Créteil, France; 2 Inserm, U955, Equipe 01, Neuropsychologie interventionnelle, Créteil, France; 3 Ecole Normale Supérieure, Département d'études Cognitives, Paris, France; 4 AP-HP, Hôpital H. Mondor- A. Chenevier, Centre de référence maladie de Huntington, Neurologie cognitive, Créteil, France; 5 AP-HP, Hôpital H. Mondor- A. Chenevier, Unité de recherche clinique, Créteil, France; 6 Hôpital de la Timone, Service de Neurologie et pathologie du mouvement, Marseille, France; 7 CHU of Amiens, Departement of Neurology, Amiens, France; 8 CHU of Angers, Centre de référence des maladies neurogénétiques, service de neurologie, Angers, France; 9 AP-HP-GHU NORD, Hôpital Avicenne, Etablissement Français du sang, Bobigny, France; 10 AP-HP, Hôpital H. Mondor- A. Chenevier, Département de Biochimie-Pharmaco-Toxicologie, Créteil, France; Centre de Recherche Jean-Pierre Aubert, FRANCE

## Abstract

**Background:**

Huntington's disease (HD) is one of several neurodegenerative disorders that have been associated with metabolic alterations. Changes in Insulin Growth Factor 1 (IGF-1) and/or insulin input to the brain may underlie or contribute to the progress of neurodegenerative processes. Here, we investigated the association over time between changes in plasma levels of IGF-1 and insulin and the cognitive decline in HD patients.

**Methods:**

We conducted a multicentric cohort study in 156 patients with genetically documented HD aged from 22 to 80 years. Among them, 146 patients were assessed at least twice with a follow-up of 3.5 ± 1.8 years. We assessed their cognitive decline using the Unified Huntington’s Disease Rating Scale, and their IGF-1 and insulin plasmatic levels, at baseline and once a year during the follow-up. Associations were evaluated using a mixed-effect linear model.

**Results:**

In the cross-sectional analysis at baseline, higher levels of IGF-1 and insulin were associated with lower cognitive scores and thus with a higher degree of cognitive impairment. In the longitudinal analysis, the decrease of all cognitive scores, except the Stroop interference, was associated with the IGF-1 level over time but not of insulin.

**Conclusions:**

IGF-1 levels, unlike insulin, predict the decline of cognitive function in HD.

## Introduction

The central nervous system plays a key role in the regulation of neuroendocrine axes and, in turn, the released neurohormones participate to the activity of different areas of the brain. On the one hand, Insulin-like growth factor-1 (IGF-1), the main effector of the somatotropic axis, has a wide range of actions in both the central and peripheral nervous system, demonstrated in various experimental models in animals and humans [1]. In particular, it has a neurotrophic and neuroprotective role in the brain. On the other hand, the role of insulin in the brain has gradually been understood from the initial concepts that the brain is insensitive to insulin to the more recent ones that insulin is an important neuromodulator, contributing to cognition, especially to the memory [2]. Moreover, a functional interrelationship between IGF-1 and insulin is known to occur. Insulin controls the expression of the IGF-1 gene, whereas insulin levels and target tissues sensitivity to insulin depend on IGF-1 levels and functions [3]. Thus, changes in IGF-1 and/or insulin input to the brain may underlie or contribute to the progress of neurodegenerative processes.

Huntington’s disease (HD) is an autosomal, dominantly inherited, progressive neurodegenerative disorder characterized by involuntary movements, especially chorea, cognitive decline, behavioural, and psychiatric disturbances, all leading to functional disability. This rare and fatal disease is caused by an abnormal elongation of a CAG repeat sequence in the gene located on the short arm of chromosome 4 and encoding the protein huntingtin [4].

Studies in animal models and human patients found evidence that endocrine and metabolic alterations accompany the progression of HD. On the one hand, decreased concentrations of the IGF binding complex and the significantly higher basal plasma levels of IGF-1 in HD patients than in controls support the hypothesis of a resistance to IGF-1 in patients with HD [5]. In addition, higher IGF-1 level at baseline was associated with greater subsequent decline in executive function and attention and may predict cognitive decline [6]. On the other hand, an impairment in insulin secretion capacity, a decrease in insulin sensitivity on both hepatic and peripheral tissues, and an increase in the level of insulin resistance have been associated with HD [7]. Given that IGF-1 is a key partner of the actions of insulin [8], insulin levels may also influence the disease cognitive impairment.

Therefore, we aim to assess the association between changes in plasma levels of IGF-1 and insulin and the cognitive decline in HD patients over time, in a prospective multicentric cohort study.

## Materials and Methods

### Participants and baseline characteristics

We report a longitudinal prospective study of manifest Huntington’s disease patients from the Predictive Biomarkers for Huntington’s disease protocol (NCT01412125), which was approved by the ethics committee of Henri Mondor Hospital (Créteil, France) in accordance with EU and French bioethics laws. All patients gave written informed consent. They were heterozygous for the *Htt* gene (> 36 CAG repeats in *mHtt*) and aware of their genetic status. They had neither other neurological conditions nor long-term experimental treatment (e.g. cell transplantation).

Data were collected from 2003 to 2009, at six centers from the French Speaking Huntington’s Disease Group (Angers: 7.7%, Créteil: 68%, Lille: 7.7%, Lyon: 0.6%, Marseille: 14.8%, Strasbourg: 1.2%), and centralized at the National Reference Centre for Huntington’s disease at Créteil.

### Clinical evaluations during follow-up

One hundred fifty six patients were assessed at baseline. One hundred and fourty six patients (94%) had at least two clinical and biological assessments so they were included in the longitudinal analysis. They were assessed once a year during the follow-up in each center. The maximum follow-up duration was of 7 years with a mean ± SD of 3.5 ± 1.8 years. The mean number of visits was 2.13 ± 1.11 (64 patients with 2 time points, 44 with 3 time points, 31 with 4 time points, 3 with 5 time points, 3 with 6 time points, and 1 patient with 7 time points). At each assessment, scores were given on the motor, cognitive, behavioural, and functional sections of the French version of the Unified Huntington’s Disease Rating Scale (UHDRS). The cognitive function was assessed using the 3 standardized tests of the UHDRS: the Stroop Interference Test, the Symbol Digit Modalities Test (SDMT), and the verbal fluency test (words starting with P, R, and V in French; the equivalent of FAS in English) [9]. The cognitive subsection takes 7 to 10 minutes to complete. Test results are reported as the raw numbers of correct answers given within the time limit for each test: 45 seconds for each Stroop Test component, 90 seconds for the SDMT, and 2 minutes for the verbal fluency test. Higher scores indicate better cognitive performance.

In addition, the patients were interviewed about medical history and completed a questionnaire on past and current symptoms. Medication use of the participants was recorded, including antipsychotics, antidepressants and tranquillizers.

### Hormonal assays

At baseline and at each follow-up clinical examination, a blood sample from each patient was collected in the morning after an overnight fast, then centralized, centrifuged and stored at -80°C at the *Etablissement Français du Sang* at Avicennes. All samples were tested in a single run using a single reagent lot at the *Département de Biochimie-Pharmaco-Toxicologie* at Henri Mondor. Kits were used according to the manufacturers’ instructions. For IGF-I and insulin-like growth factor-binding protein 3 (IGFBP-3), we used the Isys kit (IDS, Paris, France). For insulin, we used RIA kits using Glucose Analyzer 2 (Fullerton, CA, USA).

### Statistical analysis

Linear regression was performed at baseline to evaluate the associations between serum levels of IGF-1 or insulin and UHDRS cognitive scores.

In the longitudinal analysis, the individual annual slope of cognitive assessment was estimated for each task using a mixed-effect linear model with ‘‘patient” as a covariance estimate. This model takes into account the repeated measures at several time points and so the intra-individual variability. Then, we ran univariate analyses with each cognitive score as a dependent variable, and IGF-1 or insulin as independent variables in separate models. The multivariate analyses were done to adjust our models on the following potential confounding variables: age, age at onset, gender, body mass index (BMI), CAG, antipsychotics treatment, and level of education. In addition, models on IGF-1 were further adjusted for its main binding protein IGFBP-3 that regulates its activity.

Results are presented as means with their standard deviation (SD) and as mean regression slopes and their standard error (SE) or as *β* regression coefficient*s* for the linear models. A two-sided p-value ≤ 0.05 was considered statistically significant. Statistical analyses were conducted using SAS version 9.0 (SAS Institute Inc., Cary, NC).

## Results

Table 1 displays the demographic, clinical and biological characteristics of the 156 patients. Disease severity was mild to moderate in most patients (53% were stage I, 34% were stage II, 11% were stage III, and 2% were stage IV). Antipsychotics use was reported in 73 patients (46.8%). Plasma IGF-I levels ranged from 74.6 to 378.4 ng/mL, with a baseline mean of 156.4 ± 55.5 ng/mL. Plasma IGFBP-3 levels ranged from 3.0 to 7.8 μg/mL, with a baseline mean of 4.8 ± 1.1 μg/mL. Compared to the normal laboratory range, we reported 16 hyperinsulinemic (10%), 4 hypoglycaemic (3%) and 6 hyperglycaemic patients (4%).

**Table 1:** Demographic, clinical and biological features of HD patients at baseline.

Characteristics	Study population (n = 156)
**Age (years)**	47.8 ± 11.2
**Age at onset (years)**	41.6 ± 11.4
**Sex, Men/Women**	76/80
**Number of CAG repeats**	45.1 ± 3.9
**Body Mass Index (kg/m²)**	22.8 ± 3.7
**Level of education (years)**	12.0 ± 3.2
**UHDRS functional assessment**	
**Functional checklist (25–50)**	29.8 ± 5.4
**Independence scale (100 to 10)**	82.0 ± 16.0
**TFC score (13 to 0)**	9.9 ± 2.9
**UHDRS Total Motor Score (out of 124)**	35.2 ± 24.4
**UHDRS Total Behavioural Score (out of 88)**	13.3 ± 11.2
**IGF-1 (ng/mL)**	156.4 ± 55.5
**IGFBP-3 (μg/mL)**	4.8 ± 1.1
**Insulin (mUI/L)**	5.0 ± 0.9
**Glycaemia (mmol/L)**	11.5 ± 13.6

CAG: cytosine-adenine-guanine; UHDRS: Unified Huntington’s Disease Rating Scale; TFC: Total Functional Capacity; IGF-1: insulin-like growth factor-I; IGFBP-3: insulin-like growth factor -binding protein 3. Results are presented in means ± standard deviation.

### Relationship at baseline between cognitive assessments and plasma metabolic levels

In the cross-sectional analysis, cognitive scores at baseline showed moderate impairment compared with published reference values (Table 2). Both plasma IGF-1 and insulin levels were negatively associated with cognitive performance. These relationships remained significant in the multivariate analysis after adjusting for factors identified in the univariate analyses (age, sex, age at onset, number of CAG repeats, BMI, antipsychotics treatment, and level of education), and IGFBP-3 for IGF-1. The higher the levels of IGF-1 and insulin were, the more severe the cognitive decline was (IGF-1: ß Stroop word reading, -4.56, *p* = 0.06; ß Stroop color naming, -3.50, *p* = 0.04; ß Stroop interference, -5.42, *p* = 0.02; and ß SDMT, -3.58, *p* = 0.02; Insulin: ß Stroop word reading, -0.19, *p* = 0.06; ß Stroop color naming, -0.19, *p* = 0.08; ß Stroop interference, -0.22, *p* = 0.03; and ß verbal fluency test, -0.25, *p* = 0.003).

**Table 2:** Mean baseline scores and annual slopes of cognitive assessments during the follow-up in the whole cohort.

Cognitive assessment	Published reference range	Score at baseline N = 156	Annual slope ± SE N = 146	p-value
**Stroop word reading** [10]	≥ 88	61.1 ± 25.3	-4.1 ± 0.3	p<0.0001
**Stroop color naming** [10]	≥ 65	44.5 ± 20.0	-2.9 ± 0.2	p<0.0001
**Stroop interference** [10]	≥ 35	23.4 ± 13.9	-1.5 ± 0.2	p<0.0001
**SDMT** [11]	≥ 37	23.4 ± 15.5	-1.8 ± 0.2	p<0.0001
**Verbal fluency test** [12]	≥ 56	34.9 ± 22.6	-2.2 ± 0.3	p<0.0001

SDMT: Symbol Digit Modalities Test. Annual slopes are assessed using the mixed-effect linear model and adjusted for age, age at onset, CAG (cytosine-adenine-guanine) and level of education. Results are presented in means ± standard deviation. p-values of the comparison of the mean slopes to 0.

### Relationship between cognitive deterioration and evolution of the serum hormone levels

All cognitive scores declined significantly during the follow-up (Table 2). Changes in plasma levels of IGF-1 and insulin over time were not statistically significant (respectively, p = 0.8 and p = 0.4). The longitudinal relationship between cognitive deterioration and the evolution of IGF-1 and insulin levels is shown in Table 3. In the univariate analysis, the worsening of all cognitive scores, except Stroop interference, was inversely associated with plasma IGF-1 levels over time. In the multivariate analyses adjusted for age, age at onset, BMI, the number of CAG repeats, level of education, and IGFBP-3, the level of IGF-1 still negatively associated with cognitive scores. In contrast, the insulin levels over time did not show significant relation with the cognitive scores in the univariate analysis.

**Table 3:** *β* coefficients of the mixed-effect linear model for the longitudinal relationship between cognitive decline and changes of hormones plasma levels.

Clinical Data	Univariate		Multivariate
	Insulin	IGF-1	IGF-1
**Verbal Fluency**	0.04 (NS)	-0.013***	-0.011***
**SDMT**	-0.03 (NS)	-0.006**	-0.009**
**Stroop Color Naming**	-0.03 (NS)	-0.007*	-0.009***
**Stroop Word Reading**	-0.08 (NS)	-0.009*	-0.010**
**Stroop Interference**	-0.05 (NS)	NS	-

IGF-1: insulin-like growth factor-I; SDMT: Symbol Digit Modalities Test. Multivariate analyses were adjusted for potential confounders that influence cognitive decline or hormonal alterations and evaluation over time: age, age at onset, CAG (cytosine-adenine-guanine), Body Mass Index, Level of education, and IGFBP-3 (insulin-like growth factor-binding protein 3).

*p≤0.10,

**p≤0.05,

***p≤0.01.

NS: non-significant.

## Discussion

In this prospective multicentric cohort study of HD patients followed for 3.5 ± 1.8 years, we found that higher baseline plasma levels of IGF-1 and insulin were associated with greater impairment in executive functions and attention as assessed by the cognitive part of the UHDRS. These significant associations were independent from potential confounders including age, age at onset, number of CAG repeats, sex, BMI, level of education, antipsychotics treatment, and plasma IGFBP-3 for IGF-1. In the longitudinal analysis, we showed that the decline of the UHDRS cognitive scores was statistically significant over time. The worsening of all cognitive scores, except Stroop interference, was inversely associated to plasma IGF-1 levels over time, unlike insulin.

Our results confirm that the elevated plasma levels of IGF-1 observed in earlier studies of HD [5] are associated to the subsequent cognitive function deterioration seen in HD, unlike insulin. Thus, our results support the hypothesis that resistance to IGF-1 is related to the cognitive decline in patients with HD; and are in line with previous findings on HD. Aziz et al. [13] also showed that the total diurnal production of the growth hormone (GH) increases with the severity of the disease and that GH secretion was more irregular in patients with motor and a major functional impairment. Since IGF-1 controls the secretion of GH by negative feedback, the resistance to IGF-1 would result in an increase in GH, which confirms the findings above. In HD, elevated plasma levels of IGF-1 might be a cause, consequence, or compensatory counterregulation to neurodegeneration.

IGF-1 is commonly described as not only involved in brain growth, development and myelination, but also in brain plasticity and neurogenesis [14]. Normal levels of serum IGF-1 are required to maintain a broad range of brain functions. Arwert et al. [15] have demonstrated, through a meta-analysis of all studies evaluating the somatotropic axis and cognitive function in the elderly, a positive and significant relationship between the concentration of IGF-1 and cognition. The most relevant features are essentially attention, executive functions and memory. The ability of IGF-1 to promote the survival of cells has been attributed in part to the phosphatidylinositol 3-kinase/Akt pathway (PI3K/Akt). The IGF-1, through activation of the PI3K/Akt pathway, is capable of blocking cell death induced by the mutated huntingtin and reduces the formation of intranuclear inclusions. However, this route is altered in HD and probably mediates resistance to IGF-1 [16]. Furthermore, impairment in the peripheral and brain IGF-1 signalling increases the levels of Abeta and inflammatory agents in the brain [7,17] and promotes oxidative stress and deficits in energy metabolism, leading to the activation of neurodegeneration cascades [18,19].

Since the early 1990s, intervention studies in animal models have shown neuroprotective effects of IGF-1 in different models of injury and insults to the brain, administered through different routes, for example, directly through intracerebroventricular administration [20] or peripheral administration of IGF-1 [21]. Systemic IGF-1 therapy improved the cognitive function in mouse models with Alzheimer’s disease [22]. Moreover, a recent study on HD mouse models has shown that intranasal administration of recombinant human IGF-1 (rhIGF-1) improved motor activity and both peripheral and central metabolic abnormalities, along with an up regulation of Akt and an increased phosphorylation of mutant huntingtin [23]. Intranasal administration promotes rhIGF-1 delivery to the brain by its direct transport from the nasal cavity to the Central Nervous System via intraneuronal and extraneuronal pathways. Seen that IGF-1 resistance is involved in cognitive decline in HD, the control of IGF-1 may lead to a new therapeutic approach. On the model of type II diabetes characterized by insulin resistance, IGF-1 resistance in HD may be approached by direct IGF-1 administration or indirectly by increasing IGF-1 plasma levels.

Besides predicting the cognitive decline in HD, IGF-1 plasma levels over time are inversely associated to the cognitive functions, independently of other confounding factors such as age. Thus, IGF-1 is a potential candidate as a biomarker for predicting or evaluating the progress of the disease. However, its relevance for clinical application requires additional well-defined criteria and thus further investigation.

As for insulin, our cross-sectional results show that clinical hyperinsulinism is negatively associated to cognition in Huntington’s disease. Our results appear to be in line with many studies which show that hyperinsulinemia and insulin resistance are risk factors for developing dementia and affect the initiation of neuritic plaques formation [24,25]. In fact, HD patients develop diabetes mellitus about seven times more often than matched healthy control individuals [26,27].

However, despite the structural homology between IGF-1 and insulin, both at molecule and receptor levels, and despite their functional interrelationship, the insulin levels over the follow-up period did not show statistically significant associations with any of the cognitive scores changes. Thus, the control of insulin levels may not be a therapeutic target in HD. This finding opposes to other findings of neurothrophic properties of insulin infusion [28,29] and a well established efficacy in Alzheimer’s disease (AD). In AD patients, insulinic therapy could slow the dementia process. Plastino et al. [30] documented a significant slowdown in cognitive decline and stabilization on Mini-Mental Status Examination scores after a 12-month follow-up using insulinic therapy. In addition, increasing plasma insulin levels by insulin intravenous infusion, while maintaining euglycaemia, was found to facilitate recall of verbal declarative memory and enhance selective attention [31]. Intranasal insulin acutely improves memory, functional ability and synaptic plasticity of the brain in patients with early AD [32]. On the other hand, some findings demonstrate that raising peripheral insulin levels increase plasma and cerebrospinal fluid levels of norepinephrine and may modulate cognitive functions [33].

To our knowledge, this is the first study providing a long-term follow-up (up to seven years) of 146 HD patients assessing their clinical and plasmatic IGF-1 and insulin levels. Our study gives a piece of information on the impact of IGF-1 and insulin on cognition in HD. IGF-1 levels over time predict and are associated to the decline of cognitive function, unlike insulin. The temporality between IGF-1 resistance and neurodegeneration in HD remains to be explored. In addition, it would be interesting to further investigate the role of the downstream PIK3/Akt pathway in resistance to IGF-1 on one hand and in the cognitive decline in HD on the other hand.

## References

[1] Carro E, Trejo JL, Núñez A, Torres-Aleman I. Brain Repair and Neuroprotection by Serum Insulin-Like Growth Factor I. Mol Neurobiol. 2003;27: 153–162. 10.1385/MN:27:2:153 12777685

[2] McNay EC, Recknagel AK. Brain insulin signaling: a key component of cognitive processes and a potential basis for cognitive impairment in type 2 diabetes. Neurobiol Learn Mem. 2011;96: 432–442. 10.1016/j.nlm.2011.08.005 21907815 PMC3190063

[3] Torres-Aleman I. Toward a comprehensive neurobiology of IGF-I. Dev Neurobiol. 2010;70: 384–396. 10.1002/dneu.20778 20186710

[4] A novel gene containing a trinucleotide repeat that is expanded and unstable on Huntington’s disease chromosomes. The Huntington’s Disease Collaborative Research Group. Cell. 1993;72: 971–983. 8458085 10.1016/0092-8674(93)90585-e

[5] Saleh N, Moutereau S, Durr A, Krystkowiak P, Azulay J-P, Tranchant C, et al Neuroendocrine disturbances in Huntington’s disease. PloS One. 2009;4: e4962 10.1371/journal.pone.0004962 19319184 PMC2655649

[6] Saleh N, Moutereau S, Azulay J-P, Verny C, Simonin C, Tranchant C, et al High insulinlike growth factor I is associated with cognitive decline in Huntington disease. Neurology. 2010;75: 57–63. 10.1212/WNL.0b013e3181e62076 20603485

[7] Craft S. Insulin resistance and Alzheimer’s disease pathogenesis: potential mechanisms and implications for treatment. Curr Alzheimer Res. 2007;4: 147–152. 17430239 10.2174/156720507780362137

[8] Sandhu MS, Heald AH, Gibson JM, Cruickshank JK, Dunger DB, Wareham NJ. Circulating concentrations of insulin-like growth factor-I and development of glucose intolerance: a prospective observational study. Lancet Lond Engl. 2002;359: 1740–1745. 10.1016/S0140-6736(02)08655-5 12049864

[9] Unified Huntington’s Disease Rating Scale: reliability and consistency. Huntington Study Group. Mov Disord Off J Mov Disord Soc. 1996;11: 136–142. 10.1002/mds.870110204 8684382

[10] Golden C. Stroop Color and Word Test. Chicago: Stoelting; 1978.

[11] Wechsler D. Wechsler Adult Intelligence Scale–Revised Manual. New York: Psychological Corporation; 1981.

[12] Cardebat D, Doyon B, Puel M, Goulet P, Joanette Y. [Formal and semantic lexical evocation in normal subjects. Performance and dynamics of production as a function of sex, age and educational level]. Acta Neurol Belg. 1990;90: 207–217. 2124031

[13] Aziz NA, Pijl H, Frölich M, Schröder-van der Elst JP, van der Bent C, Roelfsema F, et al Growth hormone and ghrelin secretion are associated with clinical severity in Huntington’s disease. Eur J Neurol. 2010;17: 280–288. 10.1111/j.1468-1331.2009.02798.x 19845749

[14] Aberg D. Role of the growth hormone/insulin-like growth factor 1 axis in neurogenesis. Endocr Dev. 2010;17: 63–76. 10.1159/000262529 19955757

[15] Arwert LI, Deijen JB, Witlox J, Drent ML. The influence of growth hormone (GH) substitution on patient-reported outcomes and cognitive functions in GH-deficient patients: a meta-analysis. Growth Horm IGF Res Off J Growth Horm Res Soc Int IGF Res Soc. 2005;15: 47–54. 10.1016/j.ghir.2004.11.004 15701572

[16] Humbert S, Bryson EA, Cordelières FP, Connors NC, Datta SR, Finkbeiner S, et al The IGF-1/Akt pathway is neuroprotective in Huntington’s disease and involves Huntingtin phosphorylation by Akt. Dev Cell. 2002;2: 831–837. 12062094 10.1016/s1534-5807(02)00188-0

[17] Craft S. Insulin resistance syndrome and Alzheimer disease: pathophysiologic mechanisms and therapeutic implications. Alzheimer Dis Assoc Disord. 2006;20: 298–301. 17132977 10.1097/01.wad.0000213866.86934.7e

[18] Okereke OI, Kurth T, Pollak MN, Gaziano JM, Grodstein F. Fasting plasma insulin, C-peptide and cognitive change in older men without diabetes: results from the Physicians’ Health Study II. Neuroepidemiology. 2010;34: 200–207. 10.1159/000289351 20197703 PMC2883838

[19] Stolk RP, Breteler MMB, Ott A, Pols HAP, Lamberts SWJ, Grobbee DE, et al Insulin and Cognitive Function in an Elderly Population: The Rotterdam Study. Diabetes Care. 1997;20: 792–795. 10.2337/diacare.20.5.792 9135944

[20] Brywe KG, Mallard C, Gustavsson M, Hedtjärn M, Leverin A-L, Wang X, et al IGF-I neuroprotection in the immature brain after hypoxia-ischemia, involvement of Akt and GSK3beta? Eur J Neurosci. 2005;21: 1489–1502. 10.1111/j.1460-9568.2005.03982.x 15845077

[21] Fernandez AM, Gonzalez de la Vega AG, Planas B, Torres-Aleman I. Neuroprotective actions of peripherally administered insulin-like growth factor I in the injured olivo-cerebellar pathway. Eur J Neurosci. 1999;11: 2019–2030. 10336671 10.1046/j.1460-9568.1999.00623.x

[22] Torres-Aleman I. Targeting insulin-like growth factor-1 to treat Alzheimer’s disease. Expert Opin Ther Targets. 2007;11: 1535–1542. 10.1517/14728222.11.12.1535 18020976

[23] Lopes C, Ribeiro M, Duarte AI, Humbert S, Saudou F, Pereira de Almeida L, et al IGF-1 intranasal administration rescues Huntington’s disease phenotypes in YAC128 mice. Mol Neurobiol. 2014;49: 1126–1142. 10.1007/s12035-013-8585-5 24347322

[24] Luchsinger JA, Tang M-X, Shea S, Mayeux R. Hyperinsulinemia and risk of Alzheimer disease. Neurology. 2004;63: 1187–1192. 15477536 10.1212/01.wnl.0000140292.04932.87

[25] Matsuzaki T, Sasaki K, Tanizaki Y, Hata J, Fujimi K, Matsui Y, et al Insulin resistance is associated with the pathology of Alzheimer disease: the Hisayama study. Neurology. 2010;75: 764–770. 10.1212/WNL.0b013e3181eee25f 20739649

[26] Farrer LA. Diabetes mellitus in Huntington disease. Clin Genet. 1985;27: 62–67. 3156696 10.1111/j.1399-0004.1985.tb00185.x

[27] Podolsky S, Leopold NA, Sax DS. Increased frequency of diabetes mellitus in patients with Huntington’s chorea. Lancet Lond Engl. 1972;1: 1356–1358. 10.1016/s0140-6736(72)91092-6 4113563

[28] Kern W, Peters A, Fruehwald-Schultes B, Deininger E, Born J, Fehm HL. Improving influence of insulin on cognitive functions in humans. Neuroendocrinology. 2001;74: 270–280. 54694 11598383 10.1159/000054694

[29] Watson GS, Baker LD, Cholerton BA, Rhoads KW, Merriam GR, Schellenberg GD, et al Effects of insulin and octreotide on memory and growth hormone in Alzheimer’s disease. J Alzheimers Dis JAD. 2009;18: 595–602. 10.3233/JAD-2009-1165 19625744 PMC2842464

[30] Plastino M, Fava A, Pirritano D, Cotronei P, Sacco N, Sperlì T, et al Effects of insulinic therapy on cognitive impairment in patients with Alzheimer disease and diabetes mellitus type-2. J Neurol Sci. 2010;288: 112–116. 10.1016/j.jns.2009.09.022 19836029

[31] Watson GS, Craft S. Modulation of memory by insulin and glucose: neuropsychological observations in Alzheimer’s disease. Eur J Pharmacol. 2004;490: 97–113. 10.1016/j.ejphar.2004.02.048 15094077

[32] Reger MA, Watson GS, Green PS, Wilkinson CW, Baker LD, Cholerton B, et al Intranasal insulin improves cognition and modulates beta-amyloid in early AD. Neurology. 2008;70: 440–448. 10.1212/01.WNL.0000265401.62434.36 17942819

[33] Watson GS, Bernhardt T, Reger MA, Cholerton BA, Baker LD, Peskind ER, et al Insulin effects on CSF norepinephrine and cognition in Alzheimer’s disease. Neurobiol Aging. 2006;27: 38–41. 10.1016/j.neurobiolaging.2004.11.011 16298239

